# Supplementary figures and images for: Divergent RNA viruses infecting sea lice, major ectoparasites of fish
Source: PLoS Pathog. 2023 Jun 22;19(6):e1011386. doi: 10.1371/journal.ppat.1011386 (PMC10287012; doi:10.1371/journal.ppat.1011386)

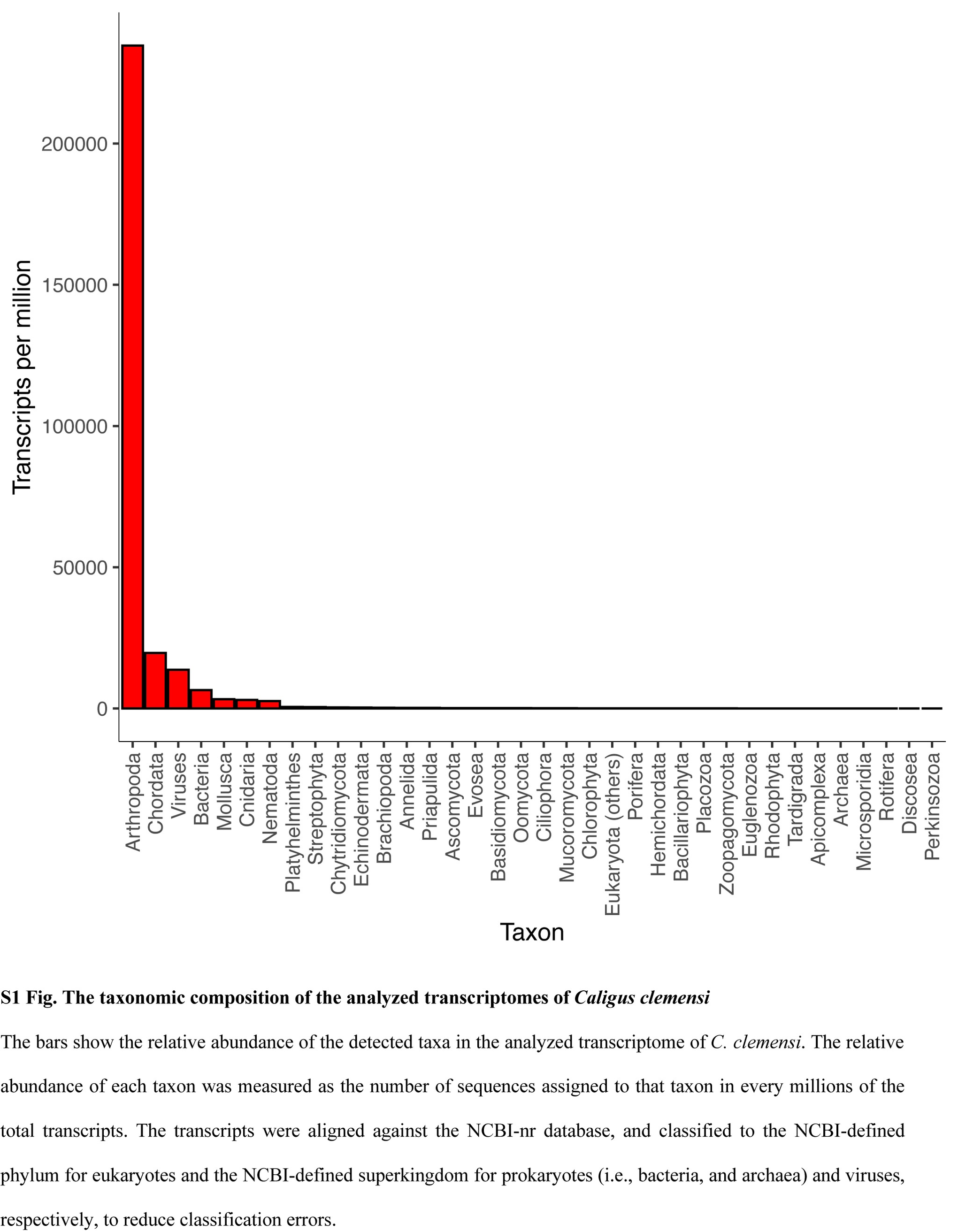

Supplement: S1 Fig — (TIF) [file ppat.1011386.s002.tif]
