# Supplementary material for: Divergent RNA viruses infecting sea lice, major ectoparasites of fish
Source: PLoS Pathog. 2023 Jun 22;19(6):e1011386. doi: 10.1371/journal.ppat.1011386 (PMC10287012; doi:10.1371/journal.ppat.1011386)
Supplement: Attachment — Submitted filename: Response_to_review_PATHOGENS-D-22-01982_20230212.docx [file ppat.1011386.s003.docx]

**PPATHOGENS-D-22-01982**

**Manuscript title:**

Pathogens of pathogens: divergent RNA viruses in sea lice (the original version)

Divergent RNA viruses infecting sea lice (the clean revised version)

**Author’s comments:**

We thank the editors and reviewers for their thoughtful comments and suggestions, which resulted in a much improved manuscript. Below we detail our responses to each of the reviewers’ comments.

**Response to Reviewer #1:**

**Summary from reviewer #1 [1]:**

Chang et al. report a detailed survey of potential RNA viruses, identified via an analysis of transcriptomes from sea lice (Caligidae). The analysis included search for both virus-derived RNA sequences and potential virus-interfering RNA molecules. The study was as comprehensive as possible and it dramatically expands the known repertoire of Caligidae-infecting RNA viruses.

The largest limitation, besides being limited by the data, available to this date, is using the NCBI CDD database for identification of RNA virus genes, RdRp in particular. The collection of profiles, available through CDD at this point, is way behind the state of the art, especially considering the recent expansion of the field, driven by large-scale metatranscriptomic studies. Some of the more exotic RdRps are poorly recognized by the CDD profiles and could have been missed. On the other hand, using the whole-CDD search (rather than a targeted search with an RdRp profile collection) provides a good protection against false positives (misidentifying DdRps and RTs as RdRps), so the author's choice could be considered as a reasonable compromise ("safe" over "bold").

**Response [1]:** We thank the reviewer for the valuable comments. We agree that compared with other available reference databases, such as NCBI-nr or virus-specific RVDB [1], NCBI-CDD contains fewer possible RdRp sequences, which might result in failure to detect some viruses encoding exotic RdRps. Therefore, as stated originally in line 514 (the original version), we first compared the meta-transcriptome assembly of *Caligus clemensi* with the entire NCBI-nr database to identify possible viral sequences, then used the CDD-annotated RdRp-encoding sequences from these virus-like sequences as probes, to search for related viruses in transcriptomes of sea lice in public databases (lines 523-527, the original version). Because of the recent expansion in genome sequences of arthropod-associated RNA viruses (AARVs), the NCBI-nr database is among the largest collection of putative coding sequences for AARVs, thus suitable for discovering potential viral sequences in sea lice. Additionally, unlike databases that only encompass viral sequences (e.g., RVDB), the nr database also includes homologs of RdRp-coding sequences from non-viral taxa. Therefore, genes like those encoding reverse-transcriptases in sea lice are less likely to be mistakenly annotated as RdRps of RNA viruses, and the potential for false-positive BLAST calls is reduced.

Annotation using CDD profiles further confirmed that sequences with significant similarity to viral RdRp-coding sequences in the nr database were from RNA viruses, given that coding sequences in the CDD are more carefully curated than those in most other sequence databases. In all the contigs with significant hits to viral RdRp sequences in the nr database, CDD also detected RdRp motifs, indicating that verification using CDD was sensitive enough to identify the divergent RdRp sequences found in our data. Another reason for using the CDD is that recent oceanic metatranscriptomic surveys reported several deeply branching RNA-virus lineages, which may represent new phyla and classes [2,3]; however, the identification of these sequences in prokaryotic genomes (e.g., CRISPR spacers), and their phylogenetic position suggests that these viruses likely infect prokaryotes or protists. Given their unsettled host link and evolutionary relationship, and that it is unlikely that these viruses infect crustaceans, we did not include them in our analysis.

In order to remove confusion, we modified the methods, as follows:

1) Line 512 (clean revised version): the subtitle was changed from 'Viral genome assembly' to 'Sequence assembly and viral sequence detection'.

2) Changes (highlighted below) were also made between lines 528 and 534 of the revision: "For the identification of viruses from publicly available transcriptomes of sea lice, ORFs from the newly discovered viruses were predicted using ORFfinder (https://www.ncbi.nlm.nih.gov/orffinder/). Notably, these newly discovered viruses were identified through the sequence alignment (BLASTx) against the entire NCBI-nr database as described above. Then, the inferred RdRp coding sequences, as well as sequences of other viral genes, were further confirmed by searching against the Conserved Domain Database (CDD) using default settings (https://www.ncbi.nlm.nih.gov/Structure/cdd/wrpsb.cgi)."

**Summary from reviewer #1 [2]:**

The results, presented by the authors, contain a surprise that gives me an uneasy feeling, the dramatic difference between the virus repertoires, discovered in Caligus rogercresseyi and Caligus clemens (Fig. 3, compare the sharp contrast between C. rogercresseyi and C. clemens to the gradual change over the life stages of C. rogercresseyi). Thee is no hard reason why their viromes could NOT differ this much, so this is not necessarily indicative of an error. Still, if possible at all, I would suggest that the authors take another look at this; maybe it is explainable by some difference in sample preparation or processing?

**Response [2]:** We agree with the reviewer that our data are not adequate to compare the taxonomic composition of RNA viruses associated with different life stages or species of sea lice. Hence, we removed the original Figures 2 and 3, as well as references to these data in the Abstract (line 41, the original version: "but increased more than two-fold..."), Results (lines 132-146, the original version), Discussion (lines 429-439, the original version), and Methods (lines 548-553, the original version), and focused on the main points of the paper: 1) the expansion of the known diversity of RNA viruses associated with sea lice; 2) the discovery of a wide array of previously unknown viruses that infect and replicate in sea lice; and 3) evidence that over evolutionary time host switching of viruses ocurred between arthropods and other eukaryotes.

**Minor issues from reviewer #1:**

The nature of the data in Figure 3 just begs for an ordination-style plot (projecting a multidimensional dataset into a low-dimensional space, e.g. a plane). This would make a nice visualization.

**Response:** We removed Figure 3, although we appreciate the reviewer's suggestion to use multivariate methods, such as PCA or NMDS, for showing the data. This would be a clearer way to show the compositional difference between the compared groups. Nevertheless, Figure 3 was intended to show the variation in the presence, absence and relative abundance of viral lineages among the libraries. However, as stated above, the cause of the variations cannot be determined with confidence, and could be caused by library preparation or environmental factors, so we removed these data from the paper.

**Response to Reviewer #2:**

**Summary from reviewer #2:**

The authors have discovered 32 new viral genomes using transcriptomic data from the species Caligus rogercresseyi, Caligus clemensi and Lepeophtheirus salmonis. For C. rogercresseyi and L. salmonis they used publicly available data, while the transcriptome of C. clemensi was sequenced by the authors. The authors expand the caligid virosphere extensively as only 5 viruses were previously known. The analysis of viRNA in the caligid host was a clever way to investigate if the viruses were actively replicating in the caligid.

Methodology is thorough and clear. However, I do think the article could benefit from a clearer presentation of the 32 new viral genomes.

**Response:** We are grateful for the positive comments from the reviewer. We revised the presentation of the genomes of the reported viruses, and provided a supplementary table, which includes the accession number, assigned taxonomy, host species, sequence length, inferred coding sequences, presence/absence of canonical viRNAs, as well as some key metrics generated from the BLAST searches using the NCBI-nr database.

**Minor issues from reviewer #2:**

It is mentioned that they have discovered 32 new viruses, but it is not easy for the reader to get a clear overview of what they have discovered. I think they are all represented in the phylogenies, and some are presented in Figure 9. But I think It would be beneficial to sort all new viral sequences in a list showing short description, sequence length, partial/complete, actively replicating based on viRNA (yes/no/not investigated) and accession number.

**Response:** We agree with the reviewer, and have provided a supplementary table to summarize the data.

**Response to Reviewer #3:**

**Summary from reviewer #3:**

The study by Chang et al reveals the diversity of RNA viruses in sea lice, an economically important group of arthropod parasites that infest fish. Only three species of sea lice were examined here, including Caligus clemensi for which the authors carry out total RNA sequencing, and Caligus rogercresseyi and Lepeophtheirus salmonis for which they analyzed the sequencing data obtained from SRA database. A total of 32 new virus species were identified, although none of them are likely to be associated with diseases/infections in fish (i.e. vector-borne virus). Based on these discoveries, the authors describe their diversity, evolutionary history, and abundance levels across different sea lice species, life stages and sex. Furthermore, they use the presence of canonical virus-derived small interfering RNAs as evidence to support the claim that these viruses, including those related to fungus or other basal eukaryotes, are associated with the principal host (i.e. see lice).

Overall, the topic is of great interest, but the study is descriptive in nature, has very limited sample size, and is flawed in study design. Furthermore, I would be very cautious and avoid general conclusions such as “cross-phylum and cross-kingdom switching between arthropods and a wide array of eukaryotes” (lines 42-45), “extensive transmission of viruses between arthropods and other eukaryotes” (lines 58-59) and “viruses that may affect sea-lice survival” (line 46), since the evidence present in this study are far from enough for such statement.

**Response:** We thank Reviewer #3, for the careful review and constructive comments. In response, we significantly revised the manuscript by removing Figures 2 and 3. As well, we added more evidence to support statements that sea lice are the hosts for the replicating viruses, and that viruses have switched between arthropods and other eukaryotes over evolutionary time. Please see our detailed response below:

**Major issues from reviewer #3 [1]:**

If this study aims to explore the RNA virus diversity in sea lice, the current sampling and sequencing is massively inadequate for such task. I would expect multiple sequencing runs representing different species and different populations of sea lice that are sampled from multiple geographic locations. However, only three species were involved in this study (lines 481-494), and among which only one (i.e. C. clemensi) is sampled and processed by the authors (30 individual in a single pool, lines 498). The rest of the sequencing data (C. rogercresseyi and L. salmonis) are downloaded from the database, and these data are limited as well. Therefore, I would expect more sequencing data from the authors to cover more species or populations of sea lice. Or, at the very least, to download more SRA data associated with sea lice. Indeed, more SRA reads are available for sea lice than those linked to TSA database.

**Response [1]:** Including more species of sea lice, or additional sequencing data would not change the interpretation of our results or the main conclusion of the study, that is many previously unknown viruses infect and replicate in sea lice. Our intention was never to exhaustively explore the diversity of viruses in sea lice across multiple geographic locations. There are more than 500 described species of sea lice, and although sampling additional species and locations would have undoubtedly revealed more viral diversity, it is beyond what this study was designed to do. What we have documented is that *Caligus clemensi*, *Caligus rogercresseyi* and *Lepeophtheirus salmonis*, the most important sea lice species affecting aquaculture and many wild fish, harbor a wide array of viruses that were previously unknown to be associated with sea lice, and that many of these viruses infect and replicate in sea lice. Moreover, many of the viral lineages occurred in the three species of sea lice, and nested within larger clades of arthropod-associated viruses, suggesting that related viruses likely infect other species of sea lice and crustaceans.

**Major issues from reviewer #3 [2]:**

The authors intend to compare virome between different life stages, gender, and species. However, there is no experimental or sequencing design for these comparisons such that I am not convinced of the results and conclusions made here (lines 132-146). First, there is no replications associated with each treatment group, and as a result no statistical significance can be derived from any of the comparisons made here. For example, in the case of C. rogercresseyi, each life-stage is represented by a single library, and therefore the variations observed here (Fig. 2) are very likely to be subject to random effect. Furthermore, comparisons are made across libraries that are derived from very different sampling and sequencing procedures (Fig. 3) which involve entirely different studies. And any of the following can have huge impact on the results: for example, whether they use the same sampling processing and storage conditions? And the same procedures for RNA extraction and library preparation? Which sequencing platforms are used? And how many individuals are mixed in the same pool, amongst others.

**Response [2]:** We appreciate these constructive criticisms and agree that the sampling design is not adequate to make inferences about the viruses associated with different life stages or sexes. As these observations were not germane to the major findings of the study, we removed Figures 2 and 3 and any mention of these data.

**Major issues from reviewer #3 [3]:**

The authors use virus-derived small RNAs (viRNA) as evidence of “active replications” of viruses within the principal host (i.e. sea lice) and claim that even fungal and plant-like viruses can infect sea lice and are likely to be “disease agents” (lines 343-344), and that they are derived from numerous “horizontal virus transmission between arthropods and fungi, nematodes, anemones, and algae” (lines 372-379). Such strong conclusions could not be made based on viRNA profiling alone. My impression, based on authors results, is that ALL RNA viruses (except for EVEs) reported here present canonical viRNAs similar to those found in other arthropods (lines 218-219). Nevertheless, I did not see viruses with a different small RNA profile such that it could be assigned to a different host group other than sea lice (i.e. parasites, symbionts, food, contaminations…)? If every single non-EVE virus is infecting sea lice, how can the authors make a distinguishment? It will be useful if the authors can provide small RNA profile of viruses that are not associated with the principal host. Otherwise, the author should consider the following scenario: (i) viruses are targeted by arthropod anti-viral system simply due to its presence within arthropod but not “actively replication”, (ii) the small RNA profile of plants or basal eukaryotes might be similar to that of arthropods, which have already been reported elsewhere.

**Response [3]:** We thank the reviewer for the comments. One of our most exciting discoveries is that viruses, whose closest classified relatives infect fungi and plants, infect and replicate in sea lice. We apologize that we did not provide enough context for a broader audience on the taxon-specificity of sRNA antiviral mechanisms. Many recent studies inferred virus-host links solely through metagenomic data, which provides circumstantial evidence that a virus is infecting a specific host, as the virus might be derived from the environment, gut contents, parasites, or symbionts. The use of viRNA overcomes this weakness.

In the present study, however, we leveraged the characteristics of the identified viRNAs to not only demonstrate that the viruses were replicating, but also that they infect sea lice, and not other eukaryotes, such as fungi or worms that may be associated with the sea lice. First, the reviewer postulates that viruses are targeted by “arthropod anti-viral system simply due to its presence within arthropod but not “actively replication”. There are no examples of which we are aware in which viRNAs are produced in response to a virus that does not infect the host producing the viRNA, which are sea-lice based on the viRNA profile. Given that there is no evidence for viRNAs being produced in response to non-infectious viruses, even in many studies of viRNAs in insects, it is reasonable to discount this as a mechanism for detecting viRNAs for viruses not known to infect arthropods. Second, the reviewer suggests that "the small RNA profile of plants or basal eukaryotes might be similar to that of arthropods". This possibility can be discounted because the siRNA antiviral response has taxon-specific features that rules out the possibility that the viRNAs might be associated with other organisms [4]. For example, the viRNAs produced by fungi have a strong 5' U preference [5], as described in previous studies and also found in an arthropod-infecting fungus [6], as well as in members of the *Hypoviridae* [7]. In contrast, in the viRNAs derived from the hypo-like virus we found in sea lice, the four bases occurred relatively evenly at the 5' position, which is consistent with viRNAs found in members of the Pancrustacea (i.e., crustaceans and insects) [8–10]. Similarly, viRNAs produced by plants [11–14], nematodes [15], and other invertebrates [16] are distinct from those of crustaceans and insects. Thus, we can say with high confidence that the viRNAs were produced by sea lice. We appreciate the reviewer’s comments because other readers may have had similar concerns, and it made us realize that we needed to strengthen our arguments in the manuscript (Discussion: lines 344-360, the clean revised version).

Additionally, in the revised version, we provided more evidence that sea lice are the bone fide hosts of the reported replicating viruses. These include the phylogenetic closeness shared between some of the viruses and EVEs (genetic chimerism resulted from sequence recombination between the parasitic element and its host) of sea lice, as well as the extremely low abundance of non-arthropod sequences detected in the transcriptome of *C. clemensi*. The latter also explains why all the identified viRNAs displayed a consistent pattern. Typically, the only detected viRNAs are from viruses that infect the primary arthropod host. A possible exception is that among 20 replicating RNA viruses identified in fruit flies, the "Twyford Virus" showed a distinct viRNA pattern [8] that resembled viRNAs generated by a fly-infecting fungus [6].

Taken together, the only reasonable explanation for our results is that the viRNAs are derived from viruses infecting sea lice. Accordingly, we added additional evidence to support our conclusions that the viRNAs we observed are from viruses that infect and replicate in sea lice, as follows: Results (lines 205-211, the clean revised version); Discussion (lines 362-372, the clean revised version); Methods (lines 577 and 586, the clean revised version).

Finally, we believe that the most parsimonious explanation for the close evolutionary relationship between some viruses replicating in sea lice and those that replicate in other eukaryotes, is host-switching over evolutionary time; these exchanges likely occurred because of the close ecological links between arthropods and these organisms. It is clear from the viRNAs and EVEs that these viruses replicate in, and have a relatively long evolutionary relationship with sea lice. Additionally, some RNA viruses infect both arthropods and other eukaryotes they interact with, such as many arthropod-borne viruses that infect vertebrates and plants. Although the evidence that we present is not definitive, it is reasonable to state that the data reveal "evidence of cross-phylum and cross-kingdom switching...", over evolutionary time.

**Minor issues from reviewer #3:**

1. Title: “pathogens of the pathogens”, there is no evidence that these viruses are pathogens, even assuming they are actively replicating within sea lice. It could be symbiotic organisms.

**Response [1]:** Although it is clear that many of these viruses infect and replicate in sea lice, we agree that pathogenesis cannot be confidently inferred. Accordingly, we have changed the title to "Divergent RNA viruses infecting sea lice". We also added a paragraph to discuss the identification of potential pathogens in sea lice (lines 460-473, the clean revised version).

2. The authors should provide a table or supplementary table which presents details of each viruses discovered in this study, such as virus names, host species, sequence length, taxonomy lineage, blast hits, abundance levels, amongst others.

**Response [2]:** We thank the reviewer for the suggestion. A supplementary table has been provided to better show the genomes of the reported viruses.

3. Fig 1. The authors should not use a schematic phylogeny to show the diversity or taxonomy groups of the newly discovered viruses. The phylogeny is not derived from the author’s data and is subject to high controversy.

**Response [3]:** Figure 1 displays the overall genetic diversity of the viruses found in sea lice, and the present phylogeny of RNA viruses that has been adopted by ICTV, where the 5 branches correspond to the five phyla of RNA viruses, respectively. The phylogeny might be controversial, but it is the one adopted by the ICTV, so is reasonable to place the newly discovered viruses in this context.

4. Line 133 and fig 2. TPM is not addable across different transcripts and should not be used as an estimate of total abundance. The authors should use RPM or other statistics instead.

**Response [4]:** We have removed this content, although to our knowledge, it is still under debate as to whether use TPM or RPM-based methods for quantifying transcripts, and comparing the estimates between libraries. TPM estimates the number of copies of a particular transcript, which would be expected to present in one million of the total transcripts [17]. Therefore, unlike RPM-based approaches, the sum of TPMs in each library is always equal, which makes it more suitable for comparing the relative abundance of specific transcripts across samples.

5. Line 142. It is unclear what “relative abundance” represents and how it is estimated.

**Response [5]:** We have removed this content. However, it was explained in the original version of the manuscript (lines 548-553).

6. Line 203. “deeply branching lineage”: poor phrasing

**Response [6]:** The phrase has been changed to "deep-branching" to be consistent with related studies (line 310, the clean revised version).

7. Lines 389-390. “ancient virus transmission between …”. It is highly speculative statement, suggest deletion.

**Response [7]:** We have removed the word "ancient" from this sentence (line 397, the clean revised version), but stand by the statement that the phylogeny represents transmission of the virus from one host to the other.

8. Lines 397-401. This is part of the results, please move this section from Discussion to Results

**Response [8]:** We appreciate the reviewer's suggestion, but decided to keep the brief section on fish viruses in the Discussion given that we did not find any known fish viruses, or closely related viral sequences in the sea-lice transcriptomes.

9. Lines 442-443. “…novel structures…” bad phrasing

**Response [9]:** the word "novel" has been replaced by "atypical" (line 433, the clean revised version).

10. There is no effort to discuss the potential pathogenicity of viruses discovered

**Response [10]:** We thank the reviewer for the suggestion. A paragraph discussing viral pathogens in sea lice has been added (lines 460-473, the clean revised version).

11. Lines 536-527. The authors should not use conserved domain database as the only database for RdRp identification, because its RdRp collection is far from comprehensive.

**Response [11]:** The CDD database was used as a verification approach. The reviewer's confusion was led by our unclear description in the Methods. We have made corresponding changes as below.

1) Line 512 (clean revised version): the subtitle was changed from 'Viral genome assembly' to 'Sequence assembly and viral sequence detection'.

2) Changes (highlighted below) were also made in methods between lines 528 and 534 of the revision: "For the identification of viruses from publicly available transcriptomes of sea lice, ORFs from the newly discovered viruses were predicted using ORFfinder (https://www.ncbi.nlm.nih.gov/orffinder/). Notably, these newly discovered viruses were identified through the sequence alignment (BLASTx) against the entire NCBI-nr database as described above. Then, the inferred RdRp coding sequences, as well as sequences of other viral genes, were further confirmed by searching against the Conserved Domain Database (CDD) using default settings (https://www.ncbi.nlm.nih.gov/Structure/cdd/wrpsb.cgi)."

12. Lines 542. Why “identical sequences were merged but marked as different viruses”?

**Response [12]:** This was following the methods used in our other projects, which aligned and trimmed a large number of viruses for phylogenetic inference. Although the viral sequences have been dereplicated prior to the sequence alignment based on sequence identity, some post-trimmed viral sequences may become completely identical, owing to the quality trimming of the alignments. However, this is not the case in the present study. To avoid further confusion, we have changed the sentence to "The trimmed alignments were manually examined in Jalview" (lines 548-549, the clean revised version).

13. All samples were sequenced in a single pool here. Although individual specimens are morphologically identified, are there any analyses of host marker gene (e.g. COI) to confirm that they belonged to the same species.

**Response [13]:** We appreciate the comment from the reviewer. DNA barcoding analyses were not performed, instead, sea lice specimens were carefully identified at Haikai Institute (https://hakai.org/), via a set of morphological characteristics unique to each life stage. Take the female adult *Caligus clemensi* as an example, the examined features include: 1) no tether; 2) very developed lunules; 3) complete segmentation in the tail; 4) deep invagination on either side of the posterior end of carapace; 5) genital segment is large, forked, and well defined; 6) gravid females will have egg strings; often hot pink in color. It has been recognized for over 10 years, that *Caligus clemensi* (primarily) and *Lepeophtheirus salmonis* are the predominant sea lice species, which infect juvenile salmon in the studied area, the Strait of Georgia, BC [18–21] (see also from Fisheries and Oceans Canada: https://www.dfo-mpo.gc.ca/aquaculture/rp-pr/parr-prra/projects-projets/2015-P-02-eng.html). Nonetheless, most of these studies focusing on sea lice biology and dynamics are also dependent on morphological identification. DNA barcoding may reveal more subtle genetic differences, but this was not pertinent to the present study, and we followed long-established protocols.

**Reference**

1.	Goodacre N, Aljanahi A, Nandakumar S, Mikailov M, Khan AS. A Reference Viral Database (RVDB) To Enhance Bioinformatics Analysis of High-Throughput Sequencing for Novel Virus Detection. mSphere. 2018;3: e00069-18. doi:10.1128/mSphereDirect.00069-18

2.	Zayed AA, Wainaina JM, Dominguez-Huerta G, Pelletier E, Guo J, Mohssen M, et al. Cryptic and abundant marine viruses at the evolutionary origins of Earth’s RNA virome. Science. 2022;376: 156–162. doi:10.1126/science.abm5847

3.	Neri U, Wolf YI, Roux S, Camargo AP, Lee B, Kazlauskas D, et al. Expansion of the global RNA virome reveals diverse clades of bacteriophages. Cell. 2022;185: 4023-4037.e18. doi:10.1016/j.cell.2022.08.023

4.	Obbard DJ, Shi M, Roberts KE, Longdon B, Dennis AB. A new lineage of segmented RNA viruses infecting animals. Virus Evolution. 2020;6: vez061. doi:10.1093/ve/vez061

5.	Donaire L, Ayllón MA. Deep sequencing of mycovirus‐derived small RNAs from Botrytis species. Mol Plant Pathol. 2016;18: 1127–1137. doi:10.1111/mpp.12466

6.	Coyle MC, Elya CN, Bronski M, Eisen MB. Entomophthovirus: an insect-derived iflavirus that infects a behavior manipulating fungal pathogen of dipterans. Microbiology; 2018 Jul. doi:10.1101/371526

7.	Lee Marzano S-Y, Neupane A, Domier L. Transcriptional and Small RNA Responses of the White Mold Fungus Sclerotinia sclerotiorum to Infection by a Virulence-Attenuating Hypovirus. Viruses. 2018;10: 713. doi:10.3390/v10120713

8.	Webster CL, Waldron FM, Robertson S, Crowson D, Ferrari G, Quintana JF, et al. The discovery, distribution, and evolution of viruses associated with *Drosophila melanogaster*. Malik HS, editor. PLoS Biol. 2015;13: e1002210. doi:10.1371/journal.pbio.1002210

9.	Remnant EJ, Shi M, Buchmann G, Blacquière T, Holmes EC, Beekman M, et al. A diverse range of novel rna viruses in geographically distinct honey bee populations. Ross SR, editor. J Virol. 2017;91: e00158-17. doi:10.1128/JVI.00158-17

10.	Rückert C, Prasad AN, Garcia-Luna SM, Robison A, Grubaugh ND, Weger-Lucarelli J, et al. Small RNA responses of *Culex* mosquitoes and cell lines during acute and persistent virus infection. Insect Biochemistry and Molecular Biology. 2019;109: 13–23. doi:10.1016/j.ibmb.2019.04.008

11.	Donaire L, Wang Y, Gonzalez-Ibeas D, Mayer KF, Aranda MA, Llave C. Deep-sequencing of plant viral small RNAs reveals effective and widespread targeting of viral genomes. Virology. 2009;392: 203–214. doi:10.1016/j.virol.2009.07.005

12.	Golyaev V, Candresse T, Rabenstein F, Pooggin MM. Plant virome reconstruction and antiviral RNAi characterization by deep sequencing of small RNAs from dried leaves. Sci Rep. 2019;9: 19268. doi:10.1038/s41598-019-55547-3

13.	Gago-Zachert S, Schuck J, Weinholdt C, Knoblich M, Pantaleo V, Grosse I, et al. Highly efficacious antiviral protection of plants by small interfering RNAs identified in vitro. Nucleic Acids Res. 2019;47: 9343–9357. doi:10.1093/nar/gkz678

14.	Lan Y, Li Y, E Z, Sun F, Du L, Xu Q, et al. Identification of virus-derived siRNAs and their targets in RBSDV-infected rice by deep sequencing. Journal of Basic Microbiology. 2018;58: 227–237. doi:10.1002/jobm.201700325

15.	Britton C, Laing R, Devaney E. Small RNAs in parasitic nematodes – forms and functions. Parasitology. 147: 855–864. doi:10.1017/S0031182019001689

16.	Waldron FM, Stone GN, Obbard DJ. Metagenomic sequencing suggests a diversity of RNA interference-like responses to viruses across multicellular eukaryotes. PLOS Genetics. 2018;14: e1007533. doi:10.1371/journal.pgen.1007533

17.	Patro R, Duggal G, Love MI, Irizarry RA, Kingsford C. Salmon: fast and bias-aware quantification of transcript expression using dual-phase inference. Nat Methods. 2017;14: 417–419. doi:10.1038/nmeth.4197

18.	Price MHH, Proboszcz SL, Routledge RD, Gottesfeld AS, Orr C, Reynolds JD. Sea Louse Infection of Juvenile Sockeye Salmon in Relation to Marine Salmon Farms on Canada’s West Coast. PLOS ONE. 2011;6: e16851. doi:10.1371/journal.pone.0016851

19.	Godwin SC, Dill LM, Reynolds JD, Krkošek M. Sea lice, sockeye salmon, and foraging competition: lousy fish are lousy competitors. Can J Fish Aquat Sci. 2015;72: 1113–1120. doi:10.1139/cjfas-2014-0284

20.	Brookson CB, Krkošek M, Hunt BPV, Johnson BT, Rogers LA, Godwin SeanC. Differential infestation of juvenile Pacific salmon by parasitic sea lice in British Columbia, Canada. Can J Fish Aquat Sci. 2020;77: 1960–1968. doi:10.1139/cjfas-2020-0160

21.	Beamish R, Wade J, Pennell W, Gordon E, Jones S, Neville C, et al. A large, natural infection of sea lice on juvenile Pacific salmon in the Gulf Islands area of British Columbia, Canada. Aquaculture. 2009;297: 31–37. doi:10.1016/j.aquaculture.2009.09.001
