# Supplementary material for: Divergent RNA viruses infecting sea lice, major ectoparasites of fish
Source: PLoS Pathog. 2023 Jun 22;19(6):e1011386. doi: 10.1371/journal.ppat.1011386 (PMC10287012; doi:10.1371/journal.ppat.1011386)
Supplement: Attachment — Submitted filename: Response_to_review_PATHOGENS-D-22-01982R1_20230407.docx [file ppat.1011386.s004.docx]

**PPATHOGENS-D-22-01982R1**

**Manuscript title:**

Divergent RNA viruses infecting sea lice (the previous version)

Divergent RNA viruses infecting sea lice, major ectoparasites of fish (the clean revised version)

**Author’s comments:**

We thank the editors and reviewers for their constructive comments and suggestions. Below we detail our responses to each of the editors' comments.

**Comment from the editors [1]:** Please retain the data on sex and age but with a caveat statement that they were not analyzed in this study but provided for future studies with increased sampling. It would be a disservice to the community not to make collected data available.

**Response [1]:** We appreciate the suggestion to keep the data on sex and age. We modified the manuscript in several ways to incorporate these changes. Accordingly, we added a section in the Results (Lines 259-272, the clean revised version), “Variation in the relative abundance of different types of viruses among sequencing libraries”, as well as added Figures 8 and 9 (the clean revised version). Figure 9 was modified as requested by the editors to "retain the data on sex and age"; only the data for *Caligus rogercresseyi* were kept; data for *C. clemensi* were removed to avoid conveying the unintended message that virome compositions were different between *C. rogercresseyi* and *C. clemensi*. In addition, we added a section to the Discussion, “Differences in viral composition among copepod sexes and life stages”, in which we emphasized that because of the lack of replications, differences in the relative abundances of virus types cannot be ascribed to differences in sex or age of the sea lice (Lines 501-510, the clean revised version), and that “Further research is needed to determine if such differences are linked to sex or age of the sea lice, methodological differences, or reflect natural variation in the composition of viral assemblages within sea lice populations”. Finally, a description of methods used for the above analyses was included, “Profiling of abundances of RNA viruses detected in transcriptomes of *C. rogercresseyi*” (Lines 625-631, the clean revised version).

**Comment from the editors [2]:** Please change the title to “Divergent RNA viruses infecting parasitic sea lice” or “Divergent RNA viruses infecting sea lice, major ectoparasites of fish” to make this aspect explicit for the broad readership without overinterpreting the available data.

**Response [2]:** We appreciate the editors' help for coming up with a suitable title; we have changed the title to "Divergent RNA viruses infecting sea lice, major ectoparasites of fish".

**Comment from the editors [3]:** All virus taxon names, for instance those ending in “-rnaviricota” in Fig. 1, should be italicized (whereas virus names should not be).

**Response [3]:** We thank the editors for pointing out our nomenclature errors. All the phyla names in Figure 1 have been italicized.

**Additional notes for References:** While reviewing the reference list, we found that three citations (#7, #8, and #42, the revised versions) were left out due to conflicts between different citation tools. We apologize for the mistake and have corrected it in the revised version. A citation for ICTV was also added (#28, the revised versions; Lines 102, 130, 323, 463, the clean revised version).

**Additional notes for Data Requirements:** We have published additional data used to generate Figs 5, 6, 8, and 9 (Lines 928-931, the clean revised version).

**Additional notes for Figure Files:** We modified Figure 2 because the accession number was missing for 'Caligus clemensi sobemo-like virus 2'. All figures were examined and regenerated using PACE.
